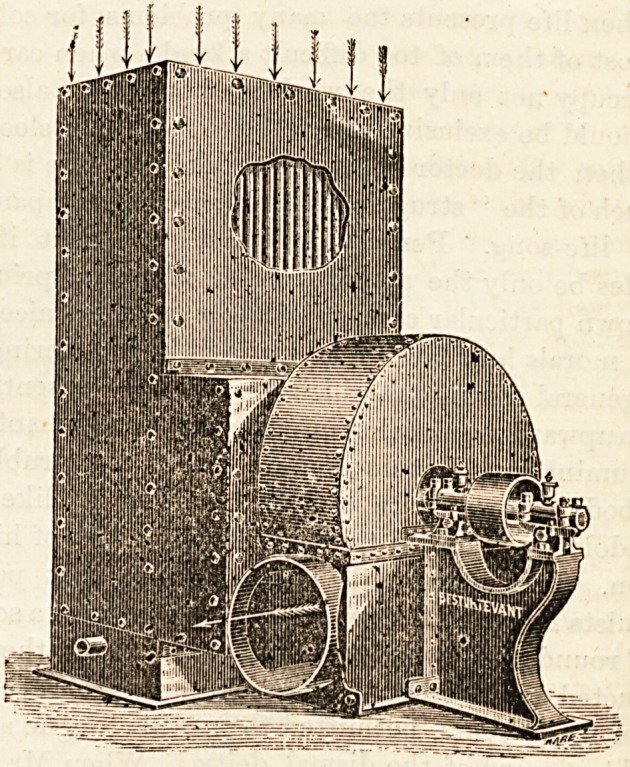# Antiseptic Ventilation for Hospitals and Sanatoriums
*Read at the Congress of the Sanitary Institute held at Worcester.


**Published:** 1889-11-02

**Authors:** S. M. Burroughs


### Antiseptic Ventilation for Hospitals and Sanatoriums.*

By S. M Burroughs.

The object of the invention or system shown is to first filter the air, then to regulate its temperature, then to propel it to any room desired, and lastly to render it antiseptic.

After a careful examination of the various systems of ventilation by forced circulation, I have selected that of the Sturtevant Blower Company, of London, as being most suitable for the application of my invention, because it can be made to blow air to any part of a building by means of sheet-iron or tin pipes.

The blower consists of a revolving fan having several blades parallel to the axis. It can be run by a steam-engine which can also be utilised for lifts, electric lights, centrifugals in laundries, mills for grinding, etc. The waste steam from the engine supplies the heat, excepting, perhaps for a large building, when it can be supplemented by live steam.

1. The air can be drawn down a chimney or shaft, and is filtered through a coarse strainer to remove the larger particles, and through finer material to take out fine dust, fog, and smoke.

2. If the air is of the right temperature it is drawn directly into the blower, but if it requires to be heated, a damper directs it into a rectangular box of sheet iron packed with tubes containing waste steam from the engine, or live steam from boiler, or both. In circulating round these tubes the air becomes heated, is drawn through the blower, and propelled through main and branch pipes to any or every part of the building.

3. If only one antiseptic or air medication be desired at one time it may be distributed from the main pipe, but a different medication can be used for each room if required.

4. A volatile antiseptic may be conveniently introduced by means of suitable mechanism, by means of which the liquid can be made to drop regularly in pure sponge or other absorbent or distributing material, from whence it is readily absorbed by the current of air. Carbolic acid creolin, pinol, terebene, pumiline, eucalyptia, thymol, or other volatile antiseptic, can be readily employed in this manner.

5. If the air is too moist or too warm, it can be both dried and cooled by causing cold water to pass through the pipes referred to instead of steam. The object of the invention is to enable hospital physicians to exactly control the temperature and to medicate the air, having previously deprived it of dust, etc.

The apparatus is not secret or patented, and can be used freely by anyone.

This apparatus constitutes the most economical system of heating buildings with which I am familiar. As a system of ventilation it is the most effectual; while for the antiseptic treatment of consumption and germ diseases, also for making antiseptic the surgical wards of hospitals, it possesses advantages over inhalers and personal appliances which interfere more or less with natural breathing.

* Read at the Congress of the Sanitary Institute held at Worcester.

## Figures and Tables

**Figure f1:**